# Tooth Loss after Jaw Curettage Surgery: Associated Factors and Potential Benefit of Splint Application

**DOI:** 10.1155/2022/7750229

**Published:** 2022-01-28

**Authors:** Zeman Qin, Haotian Cao, Yongqian Xu, Rui Chen, Zhuoying Li, Zhuoshan Huang, Meihua Zheng, Youyuan Wang, Wei-liang Chen

**Affiliations:** ^1^Department of Oral & Maxillofacial Surgery, Sun Yat-sen Memorial Hospital, Sun Yat-sen University, Guangzhou 510120, China; ^2^Department of General Dentistry, Sun Yat-sen Memorial Hospital, Sun Yat-sen University, Guangzhou 510120, China; ^3^Outpatient Department of South Campus, Sun Yat-sen Memorial Hospital, Sun Yat-sen University, Guangzhou 510275, China; ^4^Hospital of Stomatology, Sun Yat-sen University, Guangdong Provincial Key Laboratory of Stomatology, Guanghua School of Stomatology, Sun Yat-sen University, Guangzhou 510055, China

## Abstract

**Background:**

This retrospective study is aimed at (I) assessment of tooth loss and related parameters after jaw curettage of benign lesions and (II) assessment of the outcome of jaw curettage supported by splint insertion after at least six months of follow-up. *Material and Methods.* For (I), patients who had jaw curettage surgery in the Department of Oral and Maxillofacial Surgery, Sun Yat-sen Memorial Hospital, Sun Yat-sen University (Guangzhou, China) from July 2015 to June 2019 were included. For part (II), consecutive patients who came to the department from July to December 2019 that were additionally treated with dental splinting were involved in this study. Based on the patient records, age, gender, initial tooth mobility, follow-up outcome, and potential tooth loss (intra- or postoperatively) were recorded. Based on available radiographs, alveolar crest bone loss and root surface area supported by bone (RSA) were determined.

**Results:**

(I) 128 patients with 305 teeth were included, of which 40 teeth were lost (success rate 86.9%), without statistical difference in gender, age, or tooth type (*P* > 0.05). Tooth mobility, RSA, and the presence of alveolar crest bone defects were associated to tooth loss (*P* < 0.001). (II) 17 patients with a medium follow-up period of 11 months (range 9 to 13 months) were enrolled. All lesion-involving teeth supported by splint treatment at risks of loss were preserved, showing an effective tooth retention rate in 17/17 cases (74/74 teeth, success rate: 100%).

**Conclusions:**

Tooth mobility and bone loss (lesion-related and/or periodontal) are potential risk predictors for tooth loss in the first year after jaw curettage surgery. Dental splints could be recommendable for teeth involved by jaw benign lesions with little bone support.

## 1. Introduction

Apical surgery with jaw curettage is conventionally performed in treating (large) dental or nondental cysts, ameloblastoma, infections, and ambushing multiple teeth, alone, or after lesion enucleation [1–3]. After a period of 6-12 months after surgery, a high success rate of 88% survival after apical surgery is reported in literature [4]. Moreover, long-term results after 10 years reported 48.3% of teeth to be retained [5]. This success can be influenced by different factors; in general, teeth involved by or adjacent to lesions are at risk of postoperative loosening or intraoperative avulsion due to insufficient bone support [3, 6, 7]. Alongside with age, gender and tooth type (anterior vs. posterior), the remaining root surface, which is supported by bone as well as periodontal conditions (e.g., bone loss and furcation), are factors of potential relevance for preservation of teeth after apical surgery [4]. Additionally, lesions, surgical procedures, and postoperative bone remodeling often cause large defects [8, 9]. Even if the tooth survives surgical procedures and only appears slightly mobile, daily masticatory movements may prohibit periodontal healing and the tooth might have to be extracted as well [3].

Dental splints have been widely used for treating injured teeth, periodontitis-related tooth loosening, and certain types of jaw fracture, as they stabilize and maintain teeth within the periodontium, precluding further trauma and facilitating tooth retention [10–12]. Especially from the periodontal perspective, splinting did not increase the risk of tooth loss in the long-term observation and can assist tooth retention by reducing its mobility [13]. Accordingly, using a dental splint to support teeth during and following apical surgery with jaw curettage might be beneficial for preservation of these teeth.

This current retrospective cohort study was executed to (I) assess tooth loss and related parameters after jaw curettage and (II) to assess the outcome of jaw curettage because of benign lesions supported by splint insertion after at least six months of follow-up. It was hypothesized that a high success rate can be achieved in the first year after apical surgery with jaw curettage. Furthermore, the hypothesis was formulated that supporting teeth by splinting can increase the success rate in the first year of follow-up.

## 2. Methods

The current investigation was a retrospective cohort study, which followed two aims: (I) the assessment of tooth loss and related parameters after jaw curettage and (II) to assess the outcome of jaw curettage because of benign lesions supported by splint insertion after at least six months of follow-up. The study has been reviewed and approved by the Ethical Review Committee of Sun Yat-sen Memorial Hospital, Sun Yat-sen University (SYSEC-KY-KS-2019-090). Informed consent was obtained from all participants; thereby, a general informed consent for participation in research projects was provided prior to surgery. At the follow-up, a second informed consent was provided for the collection and analysis of the data in the current study. The principles outlined in the Declaration of Helsinki were followed.

### 2.1. Patients

For part (I) of the study, patients who had jaw curettage surgery in the Department of Oral and Maxillofacial Surgery, Sun Yat-sen Memorial Hospital, Sun Yat-sen University (Guangzhou, China) from July 2015 to June 2019 were included. Inclusion criteria were a surgical jaw curettage in the examination period and written informed consent. Informed consent was provided for the participation in research studies by each patient prior to surgery. Patients with an age > 79 years were excluded from the retrospective evaluation. Within part (II), consecutive patients who came to the Department of Oral and Maxillofacial Surgery, Sun Yat-sen Memorial Hospital, Sun Yat-sen University (Guangzhou, China) from July to December 2019 were involved in this study. The inclusion criteria were as follows: (i) provisional diagnosis of jaw benign lesions indicating enucleation and curettage. (ii) Lesion-involving teeth had lost bone support and therefore were at high risk of loss. (iii) Teeth were restorable and retainable that they had adequate dental tissue and crown-periodontal ligament ratio [14]. The exclusion criteria were as follows: (i) teeth with appropriate bone support, (ii) teeth unrestorable, unretainable, or deciduous, (iii) patients failed to be followed up, and (iv) patients with poor general health conditions, limiting surgical possibilities or making an adequate follow-up impossible.

Based on the medical records of participants, the following parameters were extracted: age, gender, tooth type (anterior, premolar, or molar), and mobility. Based on the available radiographs, alveolar crest defects of the lesion-involving teeth that were initially planned to preserve and loss of bone support by measuring root surface area (RSA) were determined.

### 2.2. RSA Measurement and Alveolar Bone Loss

Three experienced and calibrated periodontics specialists assessed the panoramic radiographs and/or dental cone-beam computer thomographs of patients regarding the RSA and bone loss. For calibration, the periodontics specialists evaluated the same radiographs independently, until they had an agreement of at least 80% (kappa 0.8). For every single root, the referral axis was obtained by connecting the root apex point with the midpoint of the mesial and distal alveolar crest line to calculate the intrinsic RSA (RSA_*i*_). The loss of RSA (RSA_*l*_) was calculated referring to the bone defect point of the mesial and distal line on the root. For multirooted teeth, RSA was calculated as the summery of all roots' RSA. The root was divided into *n* parts, each part was supposed to be a cylinder, the height of the cylinder was *h*, and the diameter of the cylinder was *d*, so RSA = ∑*πhd*_*i*_ = *π*∑*hd*_*i*_. If *h* was small enough, ∑h*d*_*i*_ = *S*, and RSA = *πS*^16^. Then, the loss of RSA was estimated: ratio (*r*) = RSA_*l*_/RSA_*i*_ × 100% [15, 16]. The same periodontics specialists assessed the radiographs regarding the presence or absence of alveolar crest bone loss for each tooth. Therefore, the cementoenamel junction was used as a reference point for measurement.

### 2.3. Tooth Mobility

Teeth were held by a tweezer and moved in the buccolingual direction, and the moved distance was estimated visually. Mobility was classified into grades 0, ½, 1, 1½, 2, 2½, and 3 [17] estimated by a panel of three periodontics specialists, which were calibrated as well (kappa 0.8). In case of conflicting results, a fourth periodontics specialist would be invited into discussion until consensus was reached.

### 2.4. Preoperative Preparation

After local anesthesia with mepivacaine (Scandonest, Septodont, France), all retainable lesion-involving teeth had root canal treatment. The root canal treatment was performed using of a combination with manual and rotating instruments and regularly finalized within one appointment. All root canals were filled with gutta-percha until the apex, which was checked by both endometry and radiographs. Then, the access holes were filled with composite (Filtek™ Z350 XT, 3M ESPE, U.S.A).

### 2.5. Dental Splint

The retainable loosened teeth were splinted prior to surgery with an extension to one unaffected adjacent tooth bilaterally [18]. Dental cobalt-chromium alloy wires of 0.8 mm in diameter (Shanghai Dental Material Factory, Shanghai, China) were prepared to passively fit the dental arch. The enamel bonding areas were etched with phosphoric acid (Gluma, Kulzer GmbH, Germany) and attached with wires, using adhesive (Adper™ Easy One, 3M ESPE, Germany) and composite.

### 2.6. Surgical Technique

After general anesthesia, an incision was made in the labial vestibule and a mucoperiosteal flap was carefully raised. The lesions were completely enucleated in one piece preferably and submitted for histopathologic examinations. The apexes of lesion-involving roots were resected for 2~3 mm by a round bur. Then, the bony walls and the root surfaces were curetted. Bony walls were electrocauterized to eliminate any possible remnants and then washed out with saline and curetted again to produce bleeding before watertight closure.

All patients received postsurgical instructions, including prescriptions of analgesics (ibuprofen 300 mg, three times daily, 5 days) and antibiotics (amoxicillin 500 mg, twice daily, 7 days) and keep good oral hygiene with toothbrushes, interdental brushes, and dental floss. Sutures were removed 1 week after surgery.

### 2.7. Result Evaluation

All patients were followed up for at least 6 months. Clinical and panoramic examinations were performed each time, and the healing pattern was estimated by the surgeon. At the 1-month follow-up, dental splints were removed. The response rate of a single tooth after removal of splints was graded as Table 1.

If the response rate of any tooth revealed “mostly effective,” “partially effective,” or “slightly effective,” it was splinted again. After another 1 month, splints were removed and teeth mobility was assessed again. For “ineffective” result, the tooth was extracted to avoid introducing infection.

### 2.8. Statistical Analysis

Statistical analysis was performed by using the SPSS version 22.0 to evaluate the potential outcome predictors. For testing the differences, a Mann-Whitney *U*-test was applied for metric variables. The categorical data were analyzed with chi-square or Fisher test, respectively. Difference was considered significance when *P* < 0.05.

## 3. Results

### 3.1. Study Part (I)

In total, 128 patients, who had jaw curettage surgery between July 2015 and June 2019 were included. Thereby, 305 teeth were curetted, of which 40 teeth were lost either intraoperatively or postoperatively (success rate 86.9%), without statistical difference in gender, age, or tooth type (*P* > 0.05, Table 2). Preoperative examinations demonstrated that teeth with mobility of grades ½-1, grades 1½-2, and grade 2½-3 had higher rate of getting lost (14.63%, 53.85%, and 50%, respectively) than those with grade 0 mobility (9.62%) (*P* < 0.001, Table 3). Teeth that lost bone support ratio of 60.01% to 70.00%, 70.01% to 80.00%, 80.01% to 90.00%, and 90.01% to 100% had higher rate of getting lost (26.67%, 77.78%, 75%, and 50%, respectively) than those lost less bone support ratio (*P* < 0.001). Moreover, teeth with alveolar crest defect had a 34.33% probability of getting lost, which was higher than those without alveolar crest defect (7.14%) (*P* < 0.001; Table 3).

### 3.2. Study Part (II)

A total of 31 patients visited the department with provisional diagnosis of jaw benign lesions to have enucleation and curettage from July to December 2019. Among them, 14 patients were excluded: 12 of them had CBCT evaluation of sufficient bone support around dental roots, and 2 refused to be followed up. Accordingly, a total of 17 patients with a medium follow-up period of 11 months (range 9 to 13 months) were enrolled: 7 females and 10 males, with a mean age of 29.65 (range from 14 to 47) years. Pathological findings revealed that 5 of the patients had fibrous dysplasia, 8 of them had odontogenic keratocyst, 2 of them had radicular cyst, 1 had radicular cyst, and 1 had unicystic ameloblastoma. Two of the patients had complaints of minor discomfort of teeth when loaded on the recall 1 month after surgery. All lesion-involving teeth supported by splint treatment at risk of getting lost were preserved. Therefore, an effective tooth retention rate of 17/17 cases (74/74 teeth, success rate: 100%) was achieved. Details are shown in Table 4.

## 4. Discussion

This current retrospective study revealed tooth mobility, RSA, and alveolar bone defects to be potential predictors of tooth loss after apical surgery with a jaw curettage of benign lesions. While the success rate without splinting was 86.9% after 6-12 months, splinted teeth showed a success rate of 100% in the first year after surgery.

Jaw benign lesions are common diseases which cause bone destructions [3, 19, 20]. Their conventional therapy consists of preoperative sound root canal treatment, intraoperative thorough enucleation and curettage, and postoperative watertight closure [3, 9, 21]. Surgical curettage of the bony walls is critical for therapy success and might be curative by itself [3, 9, 22]. However, teeth may fail to withstand such surgical forces and get lost due to the insufficient bone support caused by lesion-related destruction, surgical wiping out, and microfracture of thin bone plates [3, 6, 8, 9]. Thereby, loss of the buccal bone plate was found to be of relevance for tooth preservation after apical surgery [4]. The success rate in the first year after surgery that was achieved in the current retrospective study (86.9%) was comparable to another study, which investigated 281 teeth after 6-12 months and found a success rate of 88% [4]. For sure, this is just a short-term observational period; a previous German study showed a long-term survival rate of less than 50% after 10 years [5]. Furthermore, this long-term study found that retrograde endodontic treatment would increase the success rate [5]. This was not performed in the current study but might be an additional factor that should be considered in future treatment approaches. In all those considerations, it must be recognized whether it is more meaningful to analyze the sample on tooth or patient level. While analysis on tooth level is needed to estimate a prognosis, several risk predictors like smoking or general diseases as well as age always affect the whole patient.

In the current retrospective analysis, teeth with higher degree of mobility had a higher rate of getting lost. However, nearly 10% of grade 0 teeth were lost as well, and the loss of bone support and alveolar crest defect were also essential factors associated to tooth loss. It has been reported that tooth mobility is an important factor that influences the decision of tooth extraction against apical surgery [23]. Other studies did not confirm tooth mobility to be a risk predictor of tooth loss after apical surgery [4, 5]. Moreover, alveolar crest bone defects were associated to tooth loss in the current study. In absence of a full clinical periodontal status (including probing depth and clinical attachment loss), this was assessed based on X-ray diagnostics in the current study. It has been shown based on different models that biomechanical response of an apically resected tooth was affected by periodontal bone loss [24]. Additionally, probing depth and attachment loss are potential factors influencing a decision to extract teeth instead of performing apical surgery [23]. Furthermore, furcation involvement was related to reduced success rate in first year after surgery [4]. Accordingly, periodontal bone loss and loss of attachment appear a plausible risk predictor of potential tooth loss after apical surgery.

**Figure 1:**
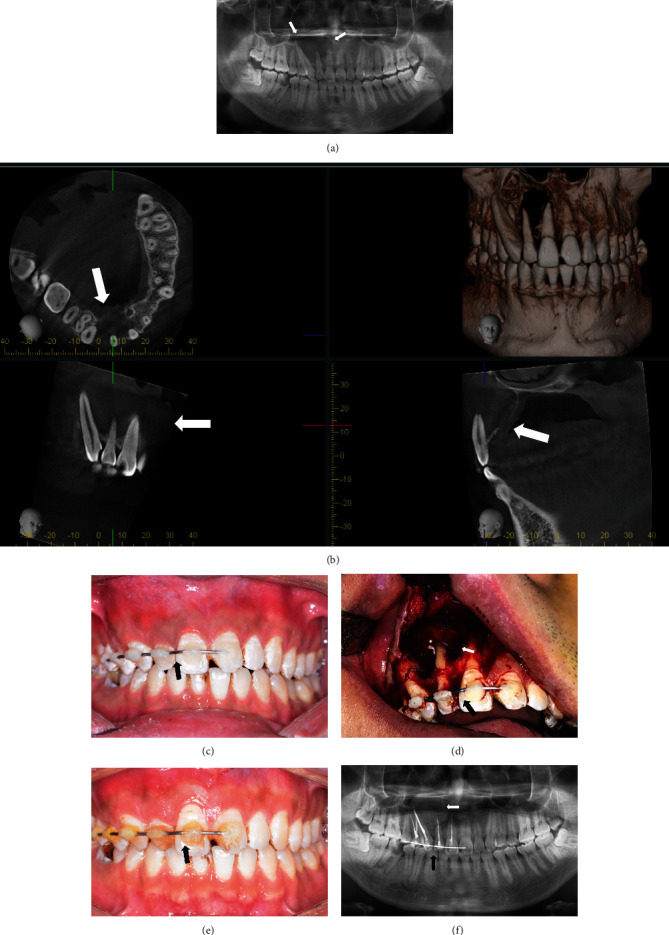
A 24-year-old male presenting with odontogenic keratocyst in the right maxilla involving 4 teeth (case 4). (a) Routine panoramic radiograph revealed a maxillary lesion with well-defined lesion, involving #11, 12, 13, and 14 (white arrow). (b) CBCT examination showed that #11, 12, 13, and 14 lost bone support (white arrow). (c) #15, 14, 13, 12, 11, and 21 were splinted before surgery (black arrow). (d) There is a large bone defect caused by lesion and surgery, and teeth had minimal bone support (white arrow). But no tooth was avulsed or excessive mobile during surgery, and the splint was fixed well on the teeth (black arrow). (e) One month after surgery, the resin of the splint had slight discoloration (black arrow). (f) Teeth were preserved, and bone defect was filled with biomaterial (white arrow).

In the second part of the analysis, this current study assessed the success rate of teeth after jaw curettage, if dental splinting supported teeth. Dental splints have been routinely used to stabilize mobile or avulsed teeth mainly caused by trauma [10, 12]. The approach to support teeth to withstand surgical forces in jaw curettage by splint application has already been considered previously [3, 6]. Some teeth had visible poor bone support intraoperatively, and it was quite a challenge for the surgeon to operate gently and curette thoroughly (Figure 1). It was noted that some preoperatively mobile teeth (Table 4) even turned stable after splinting, which proved the effectiveness of splints in helping resisting constant occlusal loading after surgery. All of the 74 splinted teeth in the 17 patients, who underwent surgery, were preserved in the first year after surgery (success rate 100%). Accordingly, the success rate of splinted teeth was higher than for the nonsplinted teeth in part (I) of the current retrospective study. Some considerations regarding splinting for apical surgery with jaw curettage should be made: surgical curettage might cause alveolar microfracture, and 4 to 8 weeks splinting would be needed for bone healing [10]. Consequently, splinting for 1 month might be a reasonable time span. This is in contrast to recent reports who recommend splints to be installed for less than 1 week to prevent root resorption or ankylosis, which were splints' major complications [10, 12]. However, this is primarily related to dental trauma, where external resorption and ankylosis might be attributed to the long extra-alveolar time of avulsed teeth rather than the duration of splinting [10, 11, 25]. Wire-composite splints are simple, fast, and inexpensive, provide stabilization, are esthetic and hygienic, do not interfere with occlusion, and allow an easy assessment of teeth on follow-up visits [11, 18]. However, adhesive bases on acid etching may damage the enamel to some extent, and it is still unclear whether splints would develop orthodontic forces [11]. Besides, wire-composite splints are predisposed to gingivitis, which is mostly reversible, but might cause marginal alveolar resorption if the fixation prolonged [11]. Thus, it is necessary to confirm the patients' good compliance and make sure that they would come back to have the splints removed before splinting starts. Therefore, splinting of teeth for jaw curettage surgery should be seen with caution and might be restricted to teeth with high risk of getting lost, especially teeth with mobility and large bone defects caused by benign lesion combined with periodontal diseases.

*Strengths and limitations*. This retrospective study was able to include a large cohort of patients with more than 300 teeth, who underwent jaw curettage. Moreover, the usage of splinting to increase the success rate within the first year of surgery appears of clinical interest. However, the study is generally limited by its retrospective design. Additionally, the short-term follow-up is an important limiting factor. Moreover, no sample size calculation was performed prior to analysis. Regarding the included patient cohort, the age span and potential presence of comorbidities are factors making the cohort quite heterogeneous. While these parameters were not assessed and considered in the current study, a potential confounding can be presumed. Information regarding any potentially associated general or syndromic diseases, especially for the splinted individuals, would have been helpful to interpret the findings. Regarding examination, a full periodontal status would have been more comprehensive and significant than only considering bone loss based on X-ray diagnostic. Thereby, it is also noticeable that the examiners were not blinded about a potential risk of bias in the current study. This could be particularly relevant in the splinted sample, whereby a success rate of 100% was found. Therefore, the conclusions need to be seen with caution in this respect, needing further validation. Moreover, besides complaints, no patient-reported outcomes were assessed in the current study. Altogether, the current study was able to show a high success rate of an additional splinting of teeth undergoing jaw curettage for benign lesions. To prove a superiority of this approach, a prospective design with a control group, an appropriate sample size, and a long-term investigation period would be necessary.

## 5. Conclusion

Following jaw curettage of benign lesions, tooth mobility and bone loss (lesion-related and/or periodontal) can be seen as risk predictors for tooth loss in the first year after surgery. Usage of a dental splint to support teeth of increased risk for getting lost might increase the success rate in the short term. Therefore, dental splints could be recommendable for teeth involved by jaw benign lesions with little bone support, which are at risk of avulsion and mobility.

## Figures and Tables

**Table 1:** The effectiveness of splinting and the result evaluation.

Effectiveness	1 month after splinting	Treatment
Effective	A grade 0 mobility or >90% reduction of mobility grade	Stop splinting
Mostly effective	A 75% to 90% reduction of mobility grade	Splint again
Partially effective	A 50% to 75% reduction of mobility grade	Splint again
Slightly effective	A 25% to 50% reduction of mobility grade	Splint again
Ineffective	A <25% reduction or even an increase of mobility grade	Stop splinting and extract the tooth

**Table 2:** Demography of included patients in part I and potential associations to tooth loss.

Category	Case number	Case number with lost teeth	Number of curetted teeth	Number of lost teeth	Percentage of teeth lost by curettage	*P* value
Demography						
*Gender*						
Male	66	22	169	22	13.02%	0.929
Female	62	18	136	18	12.68%	
*Age*						
5-9	4	0	2	0	0	0.188
10-19	21	3	61	9	14.75%	
20-29	24	6	73	9	12.33%	
30-39	24	8	56	12	21.43%	
40-49	21	1	32	1	3.13%	
50-59	12	3	41	5	12.20%	
60-69	16	1	25	3	12%	
70-79	6	1	15	1	6.67%	

**Table 3:** Clinical and radiographic findings of included teeth and potential associations to tooth loss.

Category	Number of curetted teeth	Number of lost teeth	Percentage of teeth lost by curettage	*P* value
*Type of tooth*				
Anterior teeth	98	16	16.33%	0.102
Premolar	108	17	15.74%	
Molar	99	7	7.07%	
*Tooth mobility*				
0	208	20	9.62%	**<0.001**
½-1	82	12	14.63%	
1½-2	13	7	53.85%	
2½-3	2	1	50%	
*Occasion of tooth loss*				
Intraoperatively		12	30%	-
Postoperatively		28	70%	
*RSA ratio (in %)*				
0-10.00	21	0	0	**<0.001**
10.01-20.00	30	0	0	
20.01-30.00	32	0	0	
30.01-40.00	45	1	2.22%	
40.01-50.00	48	4	8.33%	
50.01-60.00	56	2	3.57%	
60.01-70.00	45	12	26.67%	
70.01-80.00	18	14	77.78%	
80.01-90.00	8	6	75%	
90.01-100	2	1	50%	
*Alveolar crest defect*				
With	67	23	34.33%	**<0.001**
Without	238	17	7.14%	

RSA_*l*_: loss of root surface area; significant findings are highlighted in bold (*P* < 0.05).

**Table 4:** Demographic, clinical, and outcome characteristics of 5 patients had jaw curettage with dental splints.

Case/age (y)/sex	Lesion involving teeth	Teeth at risks/ratio of RSA_*l*_/alveolar crest defect (Y/N)	Pathological diagnosis	Complications	Follow-up (months)	Outcome
1/36/F	#41, 42	#41/63.10%/Y #42/59.26%/N	Fibrous dysplasia	-	13	E
2/20/M	#42, 41, 31	#41/68.75%/Y	Fibrous dysplasia	Minor discomfort of #31	12	E
3/14/F	#43, 42, 41, 31, 32, 33, 34, 35	#41/59.23%/N #42/66.67%/N#43/85%/Y #44/89.75%/Y	Fibrous dysplasia	-	12	E
4/24/M	#11, 12, 13, 14	#12/86.29%/Y #13/71.94%/Y	Odontogenic keratocyst	-	12	E
5/25/F	#21, 22, 23, 24, 25	#22/59.72%/Y #23/71.53%/N #24/65.81%/Y	Odontogenic keratocyst	-	12	E
6/38/F	#18, 17, 16, 15, 14, 13, 21, 22, 23, 24, 25, 26, 27, 28	#16/68.75%/Y #22/79.67%/Y #26/85.71%/Y #27/75%/Y	Odontogenic keratocyst	-	12	E
7/28/M	#17, 16, 15, 23, 24, 25, 26, 27	#25/70.03%/Y	Odontogenic keratocyst	-	11	E
8/41/M	#34, 35, 36, 37	#35/81.2%/N #36/78.83%/Y	Odontogenic keratocyst	-	11	E
9/27/F	#37	#37/62.53%/Y	Dentigerous cyst	Minor discomfort of #37	11	E
10/26/M	#47	#47/63.64%/Y	Radicular cyst	-	11	E
11/16/M	#43, 42, 41, 31, 32	#42/87.5%/N #41/77.76%/N #31/78.57%/Y #32/58.87%/N	Fibrous dysplasia	-	11	E
12/32/M	#37	#37/66.44%/Y	Unicystic ameloblastoma	-	11	E
13/35/F	#17, 16, 15, 14	#17/70.1%/Y	Radicular cyst	-	10	E
14/19/M	#47, 46, 45, 44, 43	#47/80.62%/N #45/64.67%/Y	Odontogenic keratocyst	-	10	E
15/47/M	#21, 22	#22/45.69%/Y	Odontogenic keratocyst	-	10	E
16/33/M	#14, 13, 12	#14/80.00%/N #13/74.31%/Y	Odontogenic keratocyst	-	9	E
17/43/F	#21, 22, 23, 24	#21/54.55%/Y #22/73.33%/Y	Fibrous dysplasia	-	9	E

Abbreviations: M: male; F: female; RSA_*l*_: loss of root surface area; Y: yes; N: no; E: effective. Tooth positions were recorded using the Federation Dentaire International (FDI) system.

## Data Availability

The data used to support the findings of this study are available from the corresponding author upon reasonable request.

## Abbreviations

CBCT: Cone-beam computer tomography
RSA: Root surface area.
